# A Metagenomic and Amplicon Sequencing Combined Approach Reveals the Best Primers to Study Marine Aerobic Anoxygenic Phototrophs

**DOI:** 10.1007/s00248-023-02220-y

**Published:** 2023-05-06

**Authors:** Carlota R. Gazulla, Ana María Cabello, Pablo Sánchez, Josep M. Gasol, Olga Sánchez, Isabel Ferrera

**Affiliations:** 1 Departament de Genètica i de Microbiologia, Universitat Autònoma de Barcelona, 08193 Bellaterra, Catalunya Spain; 2 Departament de Biologia Marina i Oceanografia, Institut de Ciències del Mar, ICM-CSIC, 08003 Barcelona, Catalunya Spain; 3 Centro Oceanográfico de Málaga, Instituto Español de Oceanografía, IEO-CSIC, 29640 Fuengirola, Málaga, Spain

**Keywords:** *pufM* gene, Primer evaluation, Metagenomics, Amplicon sequencing, AAP bacteria

## Abstract

Studies based on protein-coding genes are essential to describe the diversity within bacterial functional groups. In the case of aerobic anoxygenic phototrophic (AAP) bacteria, the *pufM* gene has been established as the genetic marker for this particular functional group, although available primers are known to have amplification biases. We review here the existing primers for *pufM* gene amplification, design new ones, and evaluate their phylogenetic coverage. We then use samples from contrasting marine environments to evaluate their performance. By comparing the taxonomic composition of communities retrieved with metagenomics and with different amplicon approaches, we show that the commonly used PCR primers are biased towards the Gammaproteobacteria phylum and some Alphaproteobacteria clades. The metagenomic approach, as well as the use of other combinations of the existing and newly designed primers, show that these groups are in fact less abundant than previously observed, and that a great proportion of *pufM* sequences are affiliated to uncultured representatives, particularly in the open ocean. Altogether, the framework developed here becomes a better alternative for future studies based on the *pufM* gene and, additionally, serves as a reference for primer evaluation of other functional genes.

**Supplementary Information:**

The online version contains supplementary material available at 10.1007/s00248-023-02220-y.

## Introduction

The open ocean microbiota consists of approximately 10^29^ organisms that perform key biogeochemical processes essential for ecosystem functioning [1]. However, only a small portion can be isolated and culture-independent techniques based on their genetic content are fundamental to study them. Indeed, the sequencing of the ribosomal 16S rRNA gene allowed the first studies on the biogeography of marine bacterial communities [2–4]. In the last decade, the development of high-throughput sequencing (HTS) methods together with worldwide oceanic surveys [5, 6] has generated a massive amount of sequencing data obtained with standardized methodologies, which has facilitated studying the marine microbiome at an unprecedent scale and has elucidated patterns of prokaryotic diversity, interactions, and connectivity around the globe (e.g., [7–13]). While most of the efforts have been performed at the whole bacterioplankton community level, the focus on specific functional groups allows the identification of microorganisms involved in a wide range of functions, such as carbon and nitrogen fixation, ammonia oxidation, or light harvesting, which are key to understand global biogeochemical cycles [12, 14]. Studies based on protein-coding genes are essential for this endeavor, since they may have experienced horizontal gene transfer (HGT) processes [15], and their phylogeny differs from the observed with the canonical ribosomal 16S rRNA gene.

A polyphyletic group that has been extensively studied in the last two decades is that of the aerobic anoxygenic phototrophic (AAP) bacteria. Their discovery in the ocean surface [16] implied a change of paradigm in our understanding of carbon cycling since they are heterotrophic organisms that can also obtain energy from light. Although they derive a fraction of their energy needs harvesting light using bacteriochlorophyll *a*, AAP bacteria are thought to be unable to fix inorganic carbon, relying thus on dissolved organic matter. Studies of the diversity of AAP communities are based on the sequencing of the *pufM* gene that encodes the M subunit of the AAP bacteria reaction center. The first versions of *pufM* primers were designed based on sequences from cultured bacteria [17–20]. A comparison of around 200 sequences from cultivated bacteria and environmental samples carried out by Yutin et al. [21] indicated that the environmental sequences of the *pufM* gene had a greater variability than the ones from cultured bacteria and these authors proposed new universal primers: pufM_uniF (forward) and pufM_uniR (reverse), hereafter called UniF and UniR, and an additional reverse primer called pufM_WAW. Although they were originally designed for marine environments, primers UniF and UniR were discarded in subsequent studies due to PCR amplification problems (e.g., [22]) and have mostly been used in freshwater ecosystems [23–25]. The combination of primers pufMF (forward), designed by Béjà et al. [20], and pufM_WAW (reverse), designed by Yutin et al. [21], was first proposed by Lehours et al. [26] on the basis of their specificity and efficiency after testing multiple primer combinations. Since then, this combination has been used in most studies analyzing AAP communities in the marine environment. Most of these studies pictured AAP communities as mainly composed by Gammaproteobacteria and Alphaproteobacteria clades [26–33]. The few studies based on metagenomics showed, however, far more diversity and a large fraction of AAP assemblages composed of members with no cultured representatives. For example, Yutin et al., [34], using the Global Ocean Sampling (GOS Expedition) metagenomic shotgun data, described several groups of AAP bacteria that were abundant in some specific areas of the ocean and that had hardly been recovered with amplicon-based methodologies. Some of these groups were also described to be abundant in samples from a Brazilian coastal bay using metagenomics [35]. A new group of AAP bacteria named “*Candidatus* Luxescamonaceae” (class Alphaproteobacteria), with a putative potential for carbon fixation, was described from the *Tara* Oceans metagenomic dataset [36]. These differences among diversity surveys based on metagenomics and amplicon-sequencing approaches are likely due to primer biases [37]. In fact, previous discussions regarding possible biases in *pufM* amplification argued that primers pufMF and pufM_WAW may overestimate some groups to the detriment of others [21, 26–29]. While metagenomics overcomes some of the PCR limitations, it generally only retrieves the most abundant members of the bacterial community. In the case of a functional group like the AAP bacteria, with a relative abundance range between 0.1 and 10% of the total bacterioplankton [38], metagenomics can limit a comprehensive knowledge of AAP diversity.

In this context, the aim of this study is to evaluate the performance of existing and newly designed primers of the *pufM* gene in marine environments. We employed several combinations of existing and novel *pufM* primers and determined their phylogenetic coverage. Then, using a selection of these primers, we compared the taxonomic composition of marine AAP communities based on amplicon sequencing vs. metagenomics. The combination of in silico tests and phylogenetic coverage analyses, together with its application to environmental samples, allow us to propose the optimal combination of primers for future studies targeting the *pufM* gene. In addition, the approach developed here can serve as reference for future studies involving primer evaluation of functional genes.

## Methods

### Building a *pufM* Database

We built a *pufM* gene database containing around 1300 sequences from isolates and metagenomes from marine environments. For that purpose, we downloaded 697 *pufM* sequences from the Genome Taxonomy Database (GTDB, https://gtdb.ecogenomic.org/, release 202) using AnnoTree [39] and then added to the database sequences from metagenomics datasets such as those from the *Tara* Oceans Expedition [6], the Malaspina Expedition [5], the GOS Expedition [34, 35], and the Blanes Bay Microbial Observatory (BBMO) [28]. The taxonomic assignation of the metagenomic sequences was based on the phylogenetic tree generated in Gazulla et al. [27]. We classified all sequences into different groups based on their class or order ranks and the phylogroups A to L, previously established by Yutin et al. [34]. These phylogroups were defined based on the *puf* operon organization and on the *pufM* gene phylogeny. Around 100 sequences could not be assigned to any phylogroup and were clustered together in the “Others” group.

### Phylogenetic coverage, primer design, and in vitro performance

To evaluate the phylogenetic coverage of the primers pufMF [20], UniF, UniR, and pufM_WAW [21], we aligned them against our *pufM* database using the *AlignTranslation* function in the *Decipher* R package [40]. We visualized the alignment with Geneious Prime® 2021.1.1. and calculated the percentage of sequences showing between 0 and 7 mismatches for each primer region within the whole database. To further evaluate these primers, we calculated in silico parameters such as the mean melting temperature, GC content, and ∆*G* values for hairpin, self-dimer and hetero-dimer formation, with the OligoAnalyzer tool from IDT (Integrated DNA Technologies, https://eu.idtdna.com/calc/analyzer). Based on the results of these analyses (Table 1), we decided to design forward primers that would combine with the reverse primer pufM_WAW, which showed good performance. The designing was carried out using the *DesignPrimers* function of the *Decipher* R package [40] and Geneious Prime® 2021.1.1. All primer proposals went through the same in silico tests as the existing primers, and their phylogenetic coverage was calculated as explained above. We came up with five candidates (Table S1) that were synthesized by ^©^Metabion International AG (https://www.metabion.com/). We performed PCR amplifications of the *pufM* gene using the different combinations of the newly designed primers as well as the primers from Béjà et al. [20] and Yutin et al., [21]. DNA from the AAP strains *Congregibacter litoralis* (Gammaproteobacteria), *Sandarakinorhabdus limnophila* (Alphaproteobacteria), and from the purple sulfur bacteria *Allochromatium vinosum* (Gammaproteobacteria), all three containing the *pufM* gene, was used as positive control, as well as several marine environmental samples from the BBMO and the Alboran Sea, which were known to contain AAP bacteria. After several attempts of amplification using various PCR conditions (see Table S2), only the following set of primers showed positive results (forward/reverse): pufMF/pufM_WAW, UniF/UniR, and pufMF_Y/pufM_WAW. The forward primer pufMF_Y was the only candidate from our designed primer proposals that successfully amplified a gene fragment. We confirmed the amplification of *pufM* fragments using Sanger sequencing, performed at the Genomics Unit of the University of Málaga, Spain (www.scai.uma.es). The following material and methods refer only to these three combinations of primers.

**Table 1:** In silico parameters of primers pufMF [20], pufM_WAW, UniF, UniR [21], and pufMF_Y (this study). Abbreviations: Tm, mean melting temperature; mismt, mismatches. The hybridization percentage refers to the percentage of sequences from our database that hybridize with 0 mismatches (0 mismt) and 0, 1, or 2 mismatches (0–2 mismt). Degenerate nucleotides are underlined

Primer name	Sequence	Length	%GC	Tm (°C)	Hybridization (%)		Hairpin*	Self- dimer*	Hetero-dimer *
					0 mismt	0–2 mismt			
pufMF	TAC GGS AAC CTG TWC TAC	18 bp	50%	62.9ºC	21.59%	69.42%	− 0.3	− 5.05	− 7.94***
pufM_WAW	AYN GCR AAC CAC CAN GCC CA	20 bp	60%	73ºC	82.50%	91.01%	1.25	− 12.32	-
UniF	GGN AAY YTN TWY TAY AAY CCN TTY CA	26 bp	36.5%	54.2ºC	94.86%	99.9%	2.56	− 12.67	− 7.22***
UniR	YCC ATN GTC CAN CKC CAR AA	20 bp	52.5%	56.9ºC	88.50%	95.75%	**	− 10.66	-
pufMF_Y	GGS AAY CTS TWY TAY AAY C	19 bp	42.1%	47.5ºC	55.5%	94.62%	2.3	− 10.53	− 8.22***

^*^Maximum value of ∆*G* (kcal/mole)

^**^No structure found for this sequence

^***^Hetero-dimer values are calculated for the following pairs of primers: pufMF/pufM_WAW, UniF/UniR, and pufMF_Y/pufM_WAW

### DNA Extraction, *pufM* Amplification, Sequencing, and Sequence Processing

To analyze the performance of these primers, we selected 17 environmental samples belonging to datasets in which AAP communities had previously been analyzed: 9 samples from a seasonal study of the BBMO [28] and 8 samples from the surface global ocean Malaspina Expedition [27] (Table S3). For this subset of samples, both amplicon (with primers pufMF/pufM_WAW) and metagenomic data were available and they represented a comprehensive picture of the diversity at a seasonal and at a spatial scale. DNA extraction and amplification of the *pufM* gene with primers pufMF/pufM_WAW was done as explained in Auladell et al. [28] and in Gazulla et al. [27] in samples from the BBMO and the Malaspina Expedition, respectively. Primers pufMF_Y/pufM_WAW and UniF/UniR were used to amplify different size fragments of the *pufM* gene following the conditions described in Supplementary Information 1. Sequencing was performed in an Illumina MiSeq sequencer (2 × 250 bp) at the Research and Testing Laboratory (http://rtlgenomics.com/). Noteworthy, amplification with primers UniF/UniR was only possible after a cleaning step performed in the sequencing house using the TaKaRa ExTaq DNA polymerase (TaKaRa Bio Inc., Shiga, Japan). Sequences of the *pufM* gene from the BBMO metagenomes were generated as described in Auladell et al. [28]. Those from the Malaspina Expedition were retrieved from the Malaspina gene catalog when annotated as any of the following: *pufM* (prokka 1.14.6 [41]), K08928 for the Kyoto Encyclopedia of Genes and Genomes orthologs (KEGG [42]), and PF00124 (Protein Families [43]). Annotations were manually curated to filter out possible false positives. The generation of the Malaspina Gene Database and its annotation is described in Supplementary Information 2 and in Sánchez et al. [44].

### Sequence Data Processing and Statistical Analyses

Each amplicon dataset was processed separately with cutadapt v1.16 [45] to remove primers and spurious sequences, and with DADA2 v1.10 [46] to differentiate exact sequence variants and remove chimeras (details in Supplementary Information 1). In total, we obtained three amplicon sequence variant (ASV) tables, each one corresponding to each primer set combination. Sample BL110412 from the BBMO dataset was discarded due to a low number of reads (103 reads in the UniF/UniR assay). In addition, to compare the performance of the different primer pairs, we joined the three amplicon datasets, by cutting all sequences to the same length (145 bp, the size of the smallest amplicon, obtained with primers UniF/UniR) and using the *mergeSequenceTables* function in DADA2, to analyze them as a single dataset. The phylogeny of the sequences was inferred using the phylogenetic tree from Gazulla et al. [27] and the Evolutionary Placement Algorithm v0.3.5 [47]. Community composition, statistical analyses and figures were performed in R v4.2.0 (R Core Team 2022) using packages *phyloseq* [48], *tidyverse* [49], *vegan* [50], and *ggplot2* [51] (see details in Supplementary Information 3).

## Results

### Phylogenetic Coverage and PCR Parameters of the Existing Primers for the Amplification of the *pufM* Gene

**Fig. 1:**
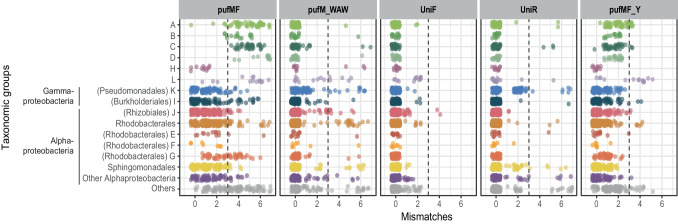
Mismatches of primers pufMF [20], pufM_WAW, UniF, and UniR, [21], and pufMF_Y (this study) for the different AAP groups defined in this study. Dashed lines separate sequences with three or more mismatches

We examined the phylogenetic coverage of existing primers pufMF [20], UniF, UniR, and pufM_WAW [21] against an in-house built database of the *pufM* gene consisting of >1300 sequences from isolates and metagenomes, by calculating the number of mismatches for each position. Then, to determine the frequency of mismatches in different AAP assemblages, we classified our database into groups according to their taxonomic rank and into the phylogroups A to L previously established by Yutin et al. [34] and commonly used in AAP diversity surveys (Fig. 1). The forward primer pufMF had the highest number of mismatches, especially for phylogroups A, B, C, D, and G, for which most of the sequences had more than three mismatches in that primer region. In total, the pufMF primer showed perfect matches to only 21.6% of the sequences in our database. This primer has been commonly paired with the reverse primer pufM_WAW, which shows a better performance, and for which most of the sequences present zero mismatches (80.4%). On the other hand, primers UniF and UniR showed a higher coverage for all the taxonomic groups, with a hybridization ratio (zero mismatches) of 94.9% for UniF and 88.5% for UniR in our database. To further evaluate these primers, we calculated in silico parameters such as the mean melting temperature, GC content, and ∆*G* values for hairpin, self-dimer, and hetero-dimer formation individually for each primer (Table 1). All primers have similar characteristics in terms of length, GC content, or degenerate nucleotides. However, the forward primer UniF stands out as the longest oligonucleotide, with a very low percentage of GC and a high number (ten) of degenerate nucleotides.

### Design of New Primers for the Amplification of the *pufM* Gene

**Fig. 2:**
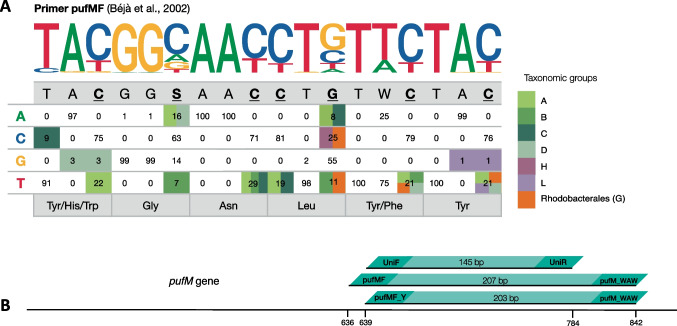
**A** Sequence logo and phylogenetic coverage of primer pufMF [20]. The table shows the percentage of sequences with each nucleotide in the different positions of the primer, based on the alignment of our *pufM* database. Nucleotides representing positions with a high number of mismatches (≥ 19% of sequences) that could be associated with specific taxonomic groups (see color legend) are in bold and underlined. **B** Schematic representation of the *pufM* gene and the primers used in this study

The design of new primers for the *pufM* gene was addressed from two perspectives: On one hand, we attempted to design primers for an upstream region that, combined with the reverse primer pufM_WAW, would generate longer amplicons and improve the taxonomic resolution. The pufM_WAW primer region is located at the end of the *pufM* gene (Fig. 2A), and since the phylogenetic coverage is high (Fig. 1; Table 1), it was a good candidate for a reverse primer. On the other hand, we intended to improve the phylogenetic coverage of the primer pufMF and the in vitro performance of primer UniF, both hybridizing in the same conserved region of the gene, by revising their design and including modifications. For this purpose, we used the nucleotide and amino acid alignment of the sequences, we analyzed the percentage of each nucleotide at each position, and we associated these changes to the different phylogroups when possible (Fig. 2B). A total of five forward primer candidates (Table S1) were tested in vitro using cultures and environmental samples and by varying the PCR conditions (annealing temperature, Mg^+2^ concentration, and primer concentration; Table S2). Three primers were designed in upstream regions of the *pufM* gene, while two were improved versions of the existing pufMF primer. We successfully obtained one forward primer, named “pufMF_Y” that combined with the existing reverse primer pufM_WAW, amplified a 203-bp fragment of the *pufM* gene, while the others were discarded due to their poor performance. This primer hybridizes in the same conserved region as primers pufMF and UniF (Fig. 2A); it improves the phylogenetic coverage of primer pufMF, and it has a lower number of degenerate nucleotides that primer UniF. Although the hybridization ratio is relatively low (55.45%) compared to primer UniF, when we consider sequences with zero, one, or two mismatches (that are likely to amplify), the hybridization ratio increases up to 95%. Additionally, we performed PCR amplification with different combinations of the existing primers. Primers pufMF/pufM_WAW and UniF/UniR amplified fragments of 207 and 145 bp, respectively. Although it has been used before in marine [52] and freshwater ecosystems [24], the combination of primers UniF/pufM_WAW did not result in positive amplification of our marine samples after several attempts under different PCR conditions.

### Oceanographic Context of the Environmental Samples Used for Primer Comparison and Sequencing Results

**Fig. 3:**
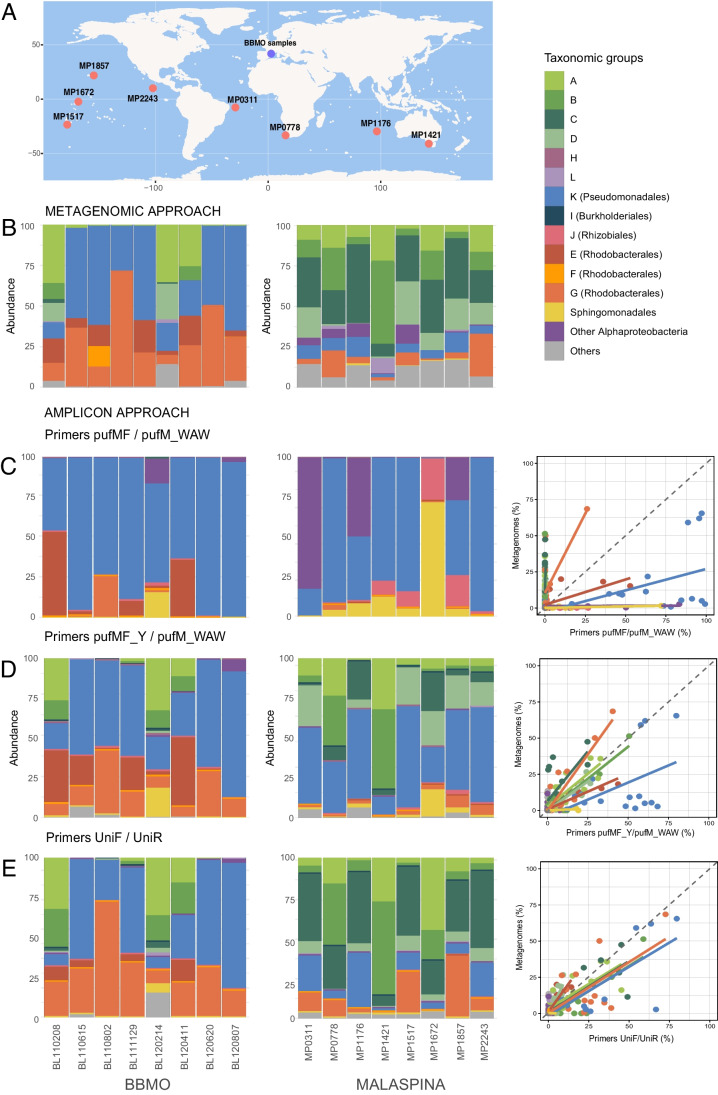
(A) Stations from the Malaspina Expedition used in this study (“MP” code). Samples from Blanes Bay Microbial Observatory (BBMO) are all from the same coastal site, yet collected at different times of the year. The code of Blanes samples indicates Blanes-year-month-day (e.g., BL110208 is from the 8th of February 2011). (B) *pufM* taxonomic composition of the BBMO left) and Malaspina (right) samples with the metagenomic approach. Below, the community composition at each station retrieved with the amplicon approach and with the different primers combinations: primers pufMF/pufM_WAW (C), pufMF_Y/pufM_WAW (D), and UniF/UniR (E). For each primer combination, and each sample, we have represented the relative abundances of the taxonomic groups retrieved with metagenomics vs. those retrieved with the amplicon approach. The dashed lines represent the 1:1 lines in which both the metagenomic and the amplicon approach would indicate the same taxonomic community composition

To test the performance of the different primers in natural samples, we compared the composition of AAP communities retrieved by metagenomics and that retrieved by amplicon sequencing using different primer combinations. Based on the in vitro results explained above, we selected primers pufMF/pufM_WAW, which have been routinely used in marine environments (e.g., [26–30, 53]), primers UniF/UniR, mainly used in freshwater ecosystems [23, 25], and the newly designed forward primer pufMF_Y with the reverse pufM_WAW. Illumina sequencing with the three primer combinations was performed for 17 samples that covered both spatial and temporal variabilities: eight open ocean samples from the Malaspina Expedition [5], from the Pacific, the Atlantic, and the Indian Oceans, and nine coastal samples from the BBMO in the Mediterranean Sea, collected at different seasons during years 2011 and 2012 [54] (Fig. 3A; Table S3). These samples were part of previous studies analyzing AAP communities by means of amplicon sequencing with primers pufMF/pufM_WAW (Malaspina samples in [27] and BBMO samples in [28]). Besides, we obtained 176 and 62 predicted genes from metagenomic assemblies associated to the *pufM* gene from the Malaspina and the BBMO metagenomic datasets, respectively. Predicted genes from Malaspina were between 101 and 1040 bp (N50 = 804 bp) in length, while genes from the BBMO were between 708 and 1044 bp long (N50 = 966 bp). Regarding the amplicon analyses, for the primer combination pufMF_Y/pufM_WAW, we obtained a total of 1904 ASVs, for the UniF/UniR, we retrieved 1294 ASVs, and with primers pufMF/pufM_WAW, we obtained a total of 418 ASVs. There were almost no shared ASVs between oceanic and coastal environments (~3%; see Table S4 for details). In terms of primer efficiency, we obtained 1.2 million reads with primers pufMF_Y/pufM_WAW, 0.5 million reads with primers UniF/UniR, and less than 0.3 million reads with primers pufMF/pufM_WAW. The high efficiency of primers pufMF_Y/pufM_WAW and the amplicon size of 203 bp (vs. 145 bp with UniF/UniR primers) led to higher values of alpha diversity for this primer combination (Fig. S1). Communities amplified with primers pufMF_Y/pufM_WAW had the highest richness (mean 173.5 ± 0.27) values, followed by primers UniF/UniR, with a significantly lower mean observed diversity (125.1 ± 2.5; Tukey test, *p* < 0.05; Fig. S1). Instead, the mean Shannon index value (pufMF_Y/pufM_WAW, 3.58; UniF/UniR, 3.36 primer) was comparable for both approaches. Primers pufMF/pufM_WAW failed to amplify many sequences as compared to the previous primers, which resulted in a significantly lower observed diversity (mean 43.9 ± 0.3; Tukey test, *p* < 0.05) and Shannon index values (mean 2.47; Tukey test, *p* < 0.05; Fig. S1).

We classified taxonomically all ASVs into 14 broad taxonomic groups, according to their order within the Alphaproteobacteria (“Rhizobiales,” “Rhodobacterales,” “Sphingomonadales” and “Other Alphaproteobacteria”) and the Gammaproteobacteria classes (“Burkholderiales” and “Pseudomonadales”, with sequences from the family Halieaceae). Besides, we also used the taxonomic groups proposed by Yutin et al. [34] –A to L– for comparison with previously published studies. Some of these phylogroups can be associated to known groups: phylogroup K contains sequences affiliated to the NOR5/OM60 clade, from family Halieaceae, order Pseudomonadales, and phylogroup I is related to the Burkholderiales order, both belonging to the Gammaproteobacteria. Phylogroup J has been associated to the Rhizobiales order, while phylogroups E, F, and G are associated to the Rhodobacterales order. Phylogroups A, B, C, D, H, and L have no taxonomically described representative. Finally, some of our ASVs could be associated to the recently described “*Candidatus* Luxescamonaceae” family. Since this family clustered within phylogroups C and D in our phylogenetic tree, we classified them as C or D depending on their position in the tree. Sequences that could not be further classified, were categorized as “Others”.

### Performance of the Primers as Compared to Metagenomics in Marine Environmental Samples

The composition of AAP communities varied largely depending on the methodology (amplicon sequencing vs. metagenomics) and the primer combination in the amplicon approaches. In the metagenomes from BBMO, phylogroup K was always present and abundant in some months, while other groups peaked at specific times of the year (Fig. 3B). This was the case of phylogroup A, whose relative abundance increased from < 1% up to 30% during winter, or the Rhodobacterales, which increased after the spring bloom. In the case of the Malaspina metagenomes, the uncultured phylogroups A, B, C, and D dominated in all samples, while Gammaproteobacteria and Alphaproteobacteria sequences were always present but scarce (Fig. 3B).

The different amplicon approaches resulted in communities with very different taxonomic composition (Fig. 3C–E; Adonis test, *p* < 0.05), especially for primers pufMF/pufM_WAW. The communities described with this primer combination were dominated by phylogroup K (Pseudomonadales), and only samples BL110208, MP0311, MP1176, and MP1672 were dominated by ASVs associated to the Alphaproteobacteria class (Fig. 3C). Noteworthy, we only retrieved three sequences with very low abundances of phylogroup C (in sample MP0778) and none from phylogroups A, B or D. Communities inferred using primers pufMF_Y/pufM_WAW were more diverse (Tukey test, *p* < 0.05) and contained more groups (Fig. 3D). Although Pseudomonadales were still prevalent, especially in coastal samples, other groups appeared as relevant, such as phylogroups A and B, and Rhodobacterales from phylogroups E and G. Likewise, primers UniF/UniR also succeeded in amplifying phylogroups A, B, C, and D. The communities retrieved with these primers were similar to those described with primers pufMF_Y/pufM_WAW, albeit with some differences. In samples from Blanes Bay, phylogroup G was predominant within the Rhodobacterales. In turn, Malaspina samples were mainly dominated by phylogroups A, B, and C, while the relative abundance of Pseudomonadales was much lower.

To further compare the performance of the three primers pairs, we plotted the relative abundance of the different taxonomic groups retrieved with each amplicon approach vs. the metagenomic approach (Fig. 3C–E) and summarized the phylogenetic coverage of the metagenomic and amplicon approaches (Fig. S2). All in all, primer pufMF failed at amplifying groups with no cultured representatives (phylogroups A, B, C, and D), while it overestimated the abundance of Gammaproteobacteria and Rhizobiales. In turn, primers pufMF_Y/pufM_WAW and UniF/UniR better reflected the composition of the communities as observed with the metagenomic approach, assumed to be less biased than amplicon approaches. Although primers pufMF_Y/pufM_WAW seemed to overestimate the abundance of Gammaproteobacteria, especially in open ocean samples (Fig. 3D), their performance was comparable to that observed with metagenomes (no statistical differences after Tukey test, *p* > 0.05; Fig. S2). In turn, the performance of primers UniF/UniR was quite good for the most abundant phylogroups.

Finally, given the differences in the taxonomic composition of samples analyzed with different primers combinations, we wanted to test whether the community structure was conserved with primers UniF/UniR and pufMF_Y/pufM_WAW. To do so, we computed Bray–Curtis dissimilarity matrices for each amplicon approach to compare the structure of communities and performed Mantel test correlations. Besides, we Hellinger-transformed the data and applied a Procrustes test to assess the statistical significance between each ordination. The matrices obtained with the primers traditionally employed in AAP diversity surveys and the primer combinations proposed in this study were highly correlated (Mantel tests; Table S5). Besides, the Procrustes tests showed no statistical differences between their spatial ordination (Table S5). For further comparison of primer’s performance, we combined all the samples into non-metric multidimensional scaling (NMDS) plots, first by merging the samples from the three amplicon approaches into one matrix (Fig. S3a) and then including the metagenomic data (Fig. S3b). Samples from coastal and open ocean environments appeared in two clear distinct clusters (dispersion between samples is statistically significant, betadisper test, *p* < 0.001; Fig. S3c) for both plots. However, the clustering of samples based on the approach (metagenomics and different primer combinations) was less clear and not significant after testing the dispersion between samples (betadisper test, *p* = 0.0793; Fig. S3d). All in all, this indicates that even though the diversity and taxonomic composition varied depending on the primer, the community structure was conserved in the three approaches, and general ecological patterns could be observed indistinctively of the used primer.

## Discussion

The study of key marker genes together with the development of the “-omics” techniques has increased our understanding of marine diversity and biogeochemical cycles [14, 55]. Both metagenomics and amplicon approaches are commonly used to target functional genes and to describe their ecological patterns. Amplicon sequencing is easy to implement, relatively cheap, and effective in capturing large numbers of sequence variants. However, due to the high sequence variability of protein-coding genes, primer biases are common and can result in the misrepresentation of the relative contribution of certain taxa. In contrast, metagenomics is PCR-free and less biased for functional gene analysis, but it generally retrieves fewer copies of marker genes and hinders the comprehensive study of functional groups with low abundances in the environment. For example, the *nifH* gene, a genetic marker of nitrogen-fixing populations, is usually studied using amplicon sequencing since the number of variants retrieved with metagenomic surveys is too low, if not undetectable [56, 57]. Likewise, most of the studies of AAP bacteria are based on the partial amplification of the *pufM* gene (e.g. [18, 20, 27–29, 31, 33, 58, 59],), while only few have approached their study based solely on metagenomics [34, 35]. Yet, the application of metagenomics allows the description of new metabolisms and new taxa in marine microbiomes, such as the discovery of new nitrogen fixation pathways in surface ocean heterotrophs [56], and the description of new AAP phylogroups previously not reported [34]. In this study, we combined both approaches to unveil the biases of existing primers for the *pufM* gene, design new ones, and test the performance of different primer combinations in a variety of marine environments.

Debate regarding primer biases in the case of the *pufM* gene is not new; it arose in studies that used the primer pair pufMF/pufM_WAW and showed a large dominance of phylogroup K (Gammaproteobacteria) in AAP communities [26–29]. Lehours et al. [26] reported > 80% of relative abundance of this phylogroup in samples from the Mediterranean Sea and considered possible primer biases favoring the amplification of the Gammaproteobacteria but disregarded that problem because the same primer pair in Arctic waters led to low abundances of phylogroup K. Still, it is likely that arctic bacterial communities have a very different composition, due to temperature differences and the effect of ice melt in salinity. Gammaproteobacteria were also predominant in samples from the coastal Blanes Bay [28, 29]. In this area, although the seasonal trends of communities retrieved by both metagenomics and amplicon sequencing had similar trends for the predominant groups, primers pufMF/pufM_WAW overestimated the contribution of phylogroup K and failed to amplify sequences of some groups that appeared in the metagenomic approach [28]. In addition to the prevalence of Gammaproteobacteria in marine AAP communities, most studies also reported the presence of members of the Alphaproteobacteria [26–29, 33, 53, 60, 61]. Our results show that for the pufMF primer, the number of mismatches of sequences affiliated to Gammaproteobacteria and some Alphaproteobacteria orders is low in comparison to the mismatches in phylogroups A, B, C, and D, which are almost absent in amplicon-based studies (Fig. 1). In fact, these phylogroups were described for the first time following the shotgun metagenomic sequencing approach of Yutin et al. [34]. They have no cultured representatives, and even though they were abundant in samples from the GOS Expedition [34], they have been barely retrieved in other studies, which might be explained by the high number of mismatches within the region of the commonly used pufMF primer (Figs. 1 and 2B). On the contrary, the universal primers UniF, UniR, and pufM_WAW [21] have a very good phylogenetic coverage (Fig. 1). While the reverse primer pufM_WAW has been extensively used in combination with pufMF, primers UniF/UniR have barely been used in marine environments and were discarded in previous studies as they repeatedly failed in amplifying under different conditions ([22, 26, 29] and authors unpublished observations). Both primers, pufMF and UniF, hybridize in the same region of the *pufM* gene (with 3-nucleotides shift between them), but UniF has 10 degenerate nucleotides, a very low GC content, and low melting temperature (Table 1), which might explain why it is problematic in vitro.

The primers designed in this study aimed at both improving the coverage of the commonly used ones and producing longer amplicons. While we did not succeed in the design of primers in the upstream region, we were able to produce an oligonucleotide with a higher hybridization ratio than pufMF, and a lower number of degenerate nucleotides than UniF, while maintaining the amplicon size of ~ 200 bp. The detailed analysis of the nucleotide composition of primer pufMF (Fig. 2B) indicates that most of the mutations in the primer region can be associated to different phylogroups and generally represent silent mutations. The only exceptions are sequences from phylogroup C and D that have a histidine and a tryptophan respectively, instead of a tyrosine in the first position (of the primer region), and sequences from phylogroup D that encode for a tyrosine instead of a phenylalanine in position five. We identified 7 nucleotides with problematic mismatches, represented in bold and underlined in Fig. 2, that were considered when redesigning the new primer pufMF_Y. Nevertheless, we cannot discard that other regions or combinations might work, nor that designs of primers that include both the *pufL* (upstream gene that encodes the L subunit of the bacterial reaction center) and the *pufM* gene might produce better tools.

Finding universally conserved regions in a functional gene is challenging, and sometimes, it is not possible to generate universal primers, as it happens, among others, with genes *nirS* and *nirK* (NO-forming nitrite reductase genes) that rely on the use of clade-specific primers [62]. Although the use of universal primers is appropriate to describe microbial communities, even perfectly matched primers can exhibit preferential amplification; thus, beyond the in silico testing, analyses with environmental samples are also important for primer evaluation [63]. To test different primer combinations, we used samples from different marine environments (coastal vs. open ocean) and from different seasons, to include the spatial and seasonal variability that exists in AAP assemblages, as reported previously [27, 28, 31, 34]. The metagenomic assay provided a bias-free representation of the most abundant components of AAP communities in samples from the BBMO and Malaspina (Fig. 3B). Even though the number of copies retrieved was low, comparing these communities to those obtained through amplicon-sequencing was the best approach to analyze the biases of each primer combination. A previous analysis with samples from the BBMO already identified discrepancies in the taxonomic composition of communities with the different methods, such as sequences from phylogroups A, B, and C that were only retrieved by metagenomics and were absent in the amplicon approach [28]. In this study, using the same extracted DNA from BBMO samples and different primer combinations, we were able to amplify sequences from these phylogroups, which in fact constitute over 50% of the relative abundance in two samples (Fig. 3D, E). Likewise, we obtained a great proportion of these groups in samples from Malaspina, which were completely missed with primers pufMF/pufM_WAW, as shown in Fig. 3C and in Gazulla et al. [27]. These results are consistent with the reports from Yutin et al., [34] and Cuadrat et al. [35], in which they describe a high proportion of sequences affiliated to phylogroups A, B, C, and D in AAP communities from different marine environments [36]. The alpha diversity estimates obtained with primers pufMF_Y/pufM_WAW and UniF/UniR (Fig. S1) surpassed by far the estimates described in samples from the BBMO [28] and the Malaspina Expedition [27] with primers pufMF/pufM_WAW. Interestingly, these previous studies had provided the most comprehensive datasets for AAP bacteria but were clearly underestimating AAP diversity.

Overall, our results indicate that the taxonomic composition of primers pufMF/pufM_WAW is biased towards phylogroup K (Pseudomonadales) (Fig. 3C), which is overestimated in almost all samples. The same happens with Sphingomonadales-like and Rhizobiales representatives and with a small cluster of other Alphaproteobacteria. Previous studies reported high abundances of Gammaproteobacteria in the Mediterranean Sea [26, 28, 29], the Baltic Sea [53], the North Pacific Ocean [60], the Arctic Sea [61], the east coast of Australia [33], or the tropical and subtropical global ocean [27]. Since these studies analyzed AAP communities with the pufMF/pufM_WAW primers, it is likely that some of these results misrepresented the real composition of AAP communities, just as we have shown for samples of the Malaspina Expedition or the BBMO. Yet, albeit the exposed primer biases, the community structure of the different approaches was conserved in different ordination tests and the matrices strongly correlated, so previously published results (e.g., the adaptation of different phylogenetic clades [31], their seasonal trends [28], or the ecological processes operating in the surface global ocean [27]) should not be discarded.

To conclude, we used an extensive *pufM* database to show the limitations of the forward primer pufMF and propose some alternatives to determine the composition and diversity of AAP communities in marine environments. We revised existing primers for the *pufM* gene in the literature, designed new ones and selected those with the best performance that were tested with environmental samples and benchmarked against metagenomics. We show that the phylogenetic coverage of primer pufMF is very low for some taxonomic groups, and, as a result, amplification with this primer is biased towards phylogroup K (Gammaproteobacteria) and some orders of the Alphaproteobacteria class. Although Gammaproteobacteria are relevant components of AAP communities, several species with no cultured representatives, like phylogroups A, B, C, and D, have been entirely underrepresented in the past and are in fact an important fraction of AAP assemblages. For future studies analyzing marine AAP bacteria, we recommend using either primers pufMF_Y/pufM_WAW or UniF/UniR, to guarantee an unbiased representation of their taxonomic composition.

## Supplementary Information

Below is the link to the electronic supplementary material.

Supplementary file1 (PDF 627 KB)

Supplementary file2 (FASTA 1206 KB)

## Data Availability

Most of the analyses were performed in R version 4.2.0 (R Core Team 2022), and code is available in the following repository: https://gitlab.com/crgazulla/analysing-pufm-primers-for-marine-aap-studies. The *pufM* database is available in the Supplementary Material. Amplicon sequences have been deposited in the NCBI Sequence Read Archive (SRA) under BioProject ID PRJNA919028. Sequences from the BBMO metagenomic dataset were published in Auladell et al., [28], and those from the Malaspina Expedition dataset are deposited under BioProject ID PRJEB52452.

## References

[1] Whitman WB, Coleman DC, Wiebe WJ (1998). Prokaryotes: the unseen majority. Proc Natl Acad Sci USA.

[2] Hagström Å, Pommier T, Rohwer F, Simu K, Stolte W, Svensson D, Zweifel UL (2002). Use of 16S ribosomal DNA for delineation of marine bacterioplankton species. Appl Environ Microbiol.

[3] Fuhrman JA, Hewson I, Schwalbach MS, Steele JA, Brown MV, Naeem S (2006) Annually reoccurring bacterial communities are predictable from ocean conditions. Proc Natl Acad Sci USA 103:13104–13109. 10.1073/pnas.0602399103 10.1073/pnas.0602399103 PMC1559760 16938845

[4] Pommier T, Canbäck L, Riemman K, Boström H, Simu K, Lundberg P, Tunlid A, Hagström Å (2007). Global patterns of diversity and community structure in marine bacterioplankton. Mol Ecol.

[5] Duarte CM (2015) Seafaring in the 21st century: the Malaspina 2010 circumnavigation expedition. Limnol Oceanogr Bull 24:11–14. 10.1002/lob.10008

[6] Karsenti E, Acinas SG, Bork P (2011). A holistic approach to marine eco-systems biology. PLoS Biol.

[7] Lima-Mendez G, Faust K, Henry N et al (2015) Determinants of community structure in the global plankton interactome. Science 348(6237):1262073. 10.1126/science.1262073 10.1126/science.1262073 25999517

[8] Sunagawa S, Coelho LP, Chaffron S (2015). Structure and function of the global ocean microbiome. Science.

[9] Mestre M, Ruiz-González C, Logares R, Duarte CM, Gasol JM, Sala MM (2018) Sinking particles promote vertical connectivity in the ocean microbiome. Proc Natl Acad Sci USA 115:E6799–E6807. 10.1073/pnas.1802470115 10.1073/pnas.1802470115 PMC6055141 29967136

[10] Logares R, Deutschmann I, Junger PC (2020). Disentangling the mechanisms shaping the surface ocean microbiota. Microbiome.

[11] Ruiz-González C, Mestre M, Estrada M (2020). Major imprint of surface plankton on deep ocean prokaryotic structure and activity. Mol Ecol.

[12] Louca S, Parfrey LW, Doebeli M (2016) Decoupling function and taxonomy in the global ocean microbiome. Science 353:1272–1277. 10.1126/science.aaf4507 10.1126/science.aaf4507 27634532

[13] Herndl GJ, Bayer B, Baltar F, Reinthaler T (2022). Prokaryotic life in the deep ocean’s water column. Ann Rev Mar Sci.

[14] Ferrera I, Sebastián M, Acinas SG, Gasol JM (2015). Prokaryotic functional gene diversity in the sunlit ocean: stumbling in the dark. Curr Opin Microbiol.

[15] Wiedenbeck J, Cohan FM (2011). Origins of bacterial diversity through horizontal genetic transfer and adaptation to new ecological niches. FEMS Microbiol Rev.

[16] Kolber ZS, van Dover CL, Niederman RA, Falkowski PG (2000). Bacterial photosynthesis in surface waters of the open ocean. Nature.

[17] Nagashima KVP, Hiraishi A, Shimada K, Matsuura K (1997). Horizontal transfer of genes coding for the photosynthetic reaction centers of purple bacteria. J Mol Evol.

[18] Achenbach LA, Carey J, Madigan MT (2001). Photosynthetic and phylogenetic primers for detection of anoxygenic phototrophs in natural environments. Appl Environ Microbiol.

[19] Tank M, Thiel V, Imhoff JF (2009). Phylogenetic relationship of phototrophic purple sulfur bacteria according to *pufL* and *pufM* genes. Intern Microbiol.

[20] Béjà O, Suzuki MT, Heidelberg JF (2002). Unsuspected diversity among marine aerobic anoxygenic phototrophs. Nature.

[21] Yutin N, Suzuki MT, Béjà O (2005). Novel primers reveal wider diversity among marine aerobic anoxygenic phototrophs. Appl Environ Microbiol.

[22] Koh EY, Phua W, Ryan KG (2011). Aerobic anoxygenic phototrophic bacteria in Antarctic sea ice and seawater. Environ Microbiol Rep.

[23] Villena-Alemany C, Mujakić I, Porcal P, Koblížek M, Piwosz K (2022). Diversity dynamics of aerobic anoxygenic phototrophic bacteria in a freshwater lake. Environ Microbiol Rep.

[24] Piwosz K, Villena-Alemany C, Mujakić I (2022). Photoheterotrophy by aerobic anoxygenic bacteria modulates carbon fluxes in a freshwater lake. ISME.

[25] Piwosz K, Vrdoljak A, Frenken T (2020). Light and primary production shape bacterial activity and community composition of aerobic anoxygenic phototrophic bacteria in a microcosm experiment. mSphere.

[26] Lehours AC, Cottrell MT, Dahan O, Kirchman DL, Jeanthon C (2010). Summer distribution and diversity of aerobic anoxygenic phototrophic bacteria in the Mediterranean Sea in relation to environmental variables. FEMS Microbiol Ecol.

[27] Gazulla CR, Auladell A, Ruiz-González C, Junger PC, Royo-Llonch M, Duarte CM, Gasol JM, Sánchez O, Ferrera I (2022). Global diversity and distribution of aerobic anoxygenic phototrophs in the tropical and subtropical oceans. Environ Microbiol.

[28] Auladell A, Sánchez P, Sánchez O, Gasol JM, Ferrera I (2019). Long-term seasonal and interannual variability of marine aerobic anoxygenic photoheterotrophic bacteria. ISME.

[29] Ferrera I, Borrego CM, Salazar G, Gasol JM (2014) Marked seasonality of aerobic anoxygenic phototrophic bacteria in the coastal NW Mediterranean Sea as revealed by cell abundance, pigment concentration and pyrosequencing of *pufM* gene. Environ Microbiol 16:2953–2965. 10.1111/1462-2920.12278 10.1111/1462-2920.12278 24131493

[30] Jeanthon C, Boeuf D, Dahan O, Le Gall F, Garczarek L, Bendif EM, Lehours AC (2011). Diversity of cultivated and metabolically active aerobic anoxygenic phototrophic bacteria along an oligotrophic gradient in the Mediterranean Sea. Biogeosciences.

[31] Lehours AC, Enault F, Boeuf D, Jeanthon C (2018). Biogeographic patterns of aerobic anoxygenic phototrophic bacteria reveal an ecological consistency of phylogenetic clades in different oceanic biomes. Sci Rep.

[32] Jiao N, Zhang Y, Zeng Y, Hong N, Liu R, Chen F, Wang P (2007). Distinct distribution pattern of abundance and diversity of aerobic anoxygenic phototrophic bacteria in the global ocean. Environ Microbiol.

[33] Bibiloni-Isaksson J, Seymour JR, Ingleton T, van de Kamp J, Bodrossy L, Brown MV (2016) Spatial and temporal variability of aerobic anoxygenic photoheterotrophic bacteria along the east coast of Australia. Environ Microbiol 18:4485–4500. 10.1111/1462-2920.13436 10.1111/1462-2920.13436 27376620

[34] Yutin N, Suzuki M, Teeling H, Weber M, Venter JC, Rusch DB, Béjà O (2007). Assessing diversity and biogeography of aerobic anoxygenic phototrophic bacteria in surface waters of the Atlantic and Pacific Oceans using the Global Ocean Sampling expedition metagenomes. Environ Microbiol.

[35] Cuadrat R, Ferrera I, Grossart HP, Dávila AMR (2016) Picoplankton bloom in global south? A high fraction of aerobic anoxygenic phototrophic bacteria in metagenomes from a coastal bay (Arraial do Cabo—Brazil). OMICS 20:76–87. 10.1089/omi.2015.0142 10.1089/omi.2015.0142 PMC4770915 26871866

[36] Graham ED, Heidelberg JF, Tully BJ (2018). Potential for primary productivity in a globally-distributed bacterial phototroph. ISME.

[37] Polz MF, Cavanaugh CM (1998). Bias in template-to-product ratios in multitemplate PCR. Appl Environ Microbiol.

[38] Koblížek M (2015). Ecology of aerobic anoxygenic phototrophs in aquatic environments. FEMS Microbiol Rev.

[39] Mendler K, Chen H, Parks DH, Lobb B, Hug LA, Doxey AC (2019). Annotree: visualization and exploration of a functionally annotated microbial tree of life. Nucleic Acids Res.

[40] Wright ES (2016). Using DECIPHER v2.0 to analyze big biological sequence data in R. R Journal.

[41] Seemann T (2014). Prokka: rapid prokaryotic genome annotation. Bioinformatics.

[42] Kanehisa M, Goto S, Sato Y, Kawashima M, Furumichi M, Tanabe M (2014). Data, information, knowledge and principle: back to metabolism in KEGG. Nucleic Acids Res.

[43] El-Gebali S, Mistry J, Bateman A (2019). The Pfam protein families database in 2019. Nucleic Acids Res.

[44] Sanchez P, Sebastián M, Pernice M et al (2023) Marine picoplankton metagenomes from eleven vertical profiles obtained by the Malaspina Expedition in the tropical and subtropical oceans. bioRxiv. 10.1101/2023.02.06.526790

[45] Martin M (2013) Cutadapt removes adapter sequences from high-throughput sequencing reads. EMBnet J 17:10. 10.14806/ej.17.1.200

[46] Callahan BJ, Mcmurdie PJ, Rosen MJ, Han AW, Johnson AJA, Holmes SP (2016). DADA2: high-resolution sample inference from Illumina amplicon data. Nat Methods.

[47] Barbera P, Kozlov AM, Czech L, Morel B, Darriba D, Flouri T, Stamakis A (2019). EPA-ng: massively parallel evolutionary placement of genetic sequences. Syst Biol.

[48] McMurdie PJ, Holmes S (2013). Phyloseq: an R package for reproducible interactive analysis and graphics of microbiome census data. PLoS One.

[49] Wickham H, Averick M, Bryan J (2019). Welcome to the Tidyverse. J Open Source Softw.

[50] Oksanen J, Simpson G, Blanchet F et al (2022) vegan: Community Ecology Package. R package version 2.6–2. https://cran.r-project.org/package=vegan. Accessed 25 Apr 2023

[51] Wickham H (2016) ggplot2: Elegant graphics for data analysis. Springer-Verlag, New York. https://ggplot2.tidyverse.org. Accessed 25 Apr 2023

[52] Fecskeová LK, Piwosz K, Šantic D, Šestanovic S, Tomaš AV, Hasunová M, Šolic M, Koblížek M (2021). Lineage-specific growth curves document large differences in response of individual groups of marine bacteria to the top-down and bottom-up controls. mSystems.

[53] Mašín M, Zdun A, Ston-Egiert J, Nausch M, Labrenz M, Moulisová V, Koblížek M (2006). Seasonal changes and diversity of aerobic anoxygenic phototrophs in the Baltic Sea. Aquatic Micro Ecol.

[54] Gasol JM, Cardelús C, Morán XAG et al (2016) Seasonal patterns in phytoplankton photosynthetic parameters and primary production at a coastal NW Mediterranean site. Sci Mar 80:63–77 10.3989/scimar.04480.06E

[55] Grossart HP, Massana R, McMahon KD, Walsh DA (2020). Linking metagenomics to aquatic microbial ecology and biogeochemical cycles. Limnol Oceanogr.

[56] Delmont TO, Quince C, Shaiber A (2018). Nitrogen-fixing populations of Planctomycetes and Proteobacteria are abundant in surface ocean metagenomes. Nat Microbiol.

[57] Cornejo-Castillo FM, Zehr JP (2021). Intriguing size distribution of the uncultured and globally widespread marine non-cyanobacterial diazotroph Gamma-A. ISME.

[58] Oz A, Sabehi G, Koblízek M, Massana R, Béjà O (2005) *Roseobacter*-like bacteria in Red and Mediterranean Sea aerobic anoxygenic photosynthetic populations. Appl Environ Microbiol 71:344–353. 10.1128/AEM.71.1.344-353.2005 10.1128/AEM.71.1.344-353.2005 PMC544225 15640208

[59] Karr EA, Sattley WM, Jung DO, Madigan MT, Achenbach LA (2003). Remarkable diversity of phototrophic purple bacteria in a permanently frozen Antarctic lake. Appl Environ Microbiol.

[60] Boeuf D, Cottrell MT, Kirchman DL, Lebaron P, Jeanthon C (2013). Summer community structure of aerobic anoxygenic phototrophic bacteria in the western Arctic Ocean. FEMS Microbiol Ecol.

[61] Lehours A, Jeanthon C (2015). The hydrological context determines the beta-diversity of aerobic anoxygenic phototrophic bacteria in European Arctic seas but does not favor endemism. Front Microbiol.

[62] Bonilla-Rosso G, Wittorf L, Jones CM, Hallin S (2018). Design and evaluation of primers targeting genes encoding NO-forming nitrite reductases: implications for ecological inference of denitrifying communities. Sci Rep.

[63] Parada AE, Needham DM, Fuhrman JA (2016). Every base matters: assessing small subunit rRNA primers for marine microbiomes with mock communities, time series and global field samples. Environ Microbiol.

