# Supplementary material for: A Metagenomic and Amplicon Sequencing Combined Approach Reveals the Best Primers to Study Marine Aerobic Anoxygenic Phototrophs
Source: Microb Ecol. 2023 May 6;86(3):2161–72. doi: 10.1007/s00248-023-02220-y (PMC10497671; doi:10.1007/s00248-023-02220-y)
Supplement: Supplementary file 1 — Supplementary file1 (PDF 627 KB) [file 248_2023_2220_MOESM1_ESM.pdf]

A metagenomic and amplicon sequencing combined approach reveals the best primers to study aerobic anoxygenic phototrophs in the marine environment

Carlota R. Gazulla[1,2], Ana María Cabello[3], Pablo Sánchez[2], Josep M. Gasol[2], Olga Sánchez[1], Isabel Ferrera[3]

Supplementary Material:

Table S1. Primer candidates designed for *pufM* amplification

Table S2. Variation of the PCR conditions for the primer candidates

Table S3. Oceanographic context of the samples used in this study

Table S4. Summary of the ASVs retrieved with each approach

Table S5. Mantel tests and Procrustes tests results

Figure S1. Alpha diversity

Figure S2. Relative abundance of taxonomic groups retrieved with the amplicon and metagenomes approaches.

Figure S3. Non-metric multidimensional scaling (NMDS) analyses.

Supplementary Information 1. PCR conditions for primers pufMF_Y/pufM_WAW and UniF/UniR

Supplementary Information 2. Metagenomic analyses of the Malaspina Expedition samples

Table S1. Primer candidates designed for *pufM* amplification. Abbreviations; Tm = mean melting temperature, mismt = mismatches.

**Primer candidate name**	**Sequence**	**Length (bp)**	**Hybridization(%)**		**Tm (ºC)**	**GC %**
			**0 mismt**	**0-2 mismt**		
pufMF_V	5’-GGS AAY CTS TWY TAY AAY CCV T-3’	22	54.3	93.3	53.6	44
pufMF_Y	5’-GGS AAY CTS TWY TAY AAY C-3’	19	55.5	94.62	47.5	42.1
pufM_F1A	5’- GAR GCN GTK CCN TWY GGN RT-3'	20	61	94	58.9	57.5
pufM_F1B	5’- GAR GCN GTK CCN TWY GGN AT-3'	20	57	93	58.9	57.5
pufM_F2	5’- TGG GCN TTY GCN DSN GCV AT-3'	20	60	75.5	62.9	60

Table S2. PCR conditions for the primer candidates pufMF_V, pufMF_Y, pufM_F1A, pufM_F1B, pufM_F2 and pufM_WAW from Bejá et al., (2002), and UniF and UniR from Yutin et al., (2005). Only two combinations were successful in the amplification of the *pufM* gene fragment, the optimal PCR conditions of both assays appear in bold and underlined.

**Forward primer**	**Reverse primer**	**Temperature (ºC)**	**Mg^{+2} (mM)**	**Primer concentration (µM)**	**Amplicon length (bp)**	**Amplification results**
pufMF_V	pufM_WAW	51ºC – 63ºC	2 – 2.5	0.2 – 0.8	203 bp	No product
**<u>pufMF_Y</u>**	**<u>pufM_WAW</u>**	**<u>51ºC</u>** – 63ºC	2 – **<u>2.5</u>**	0.2 – **<u>0.8</u>**	203 bp	**<u>Amplification</u>**
pufM_F1A	pufM_WAW	50ºC – 59ºC	2 – 2.5	0.2 – 0.8	265 bp	No product
pufM_F1B	pufM_WAW	50ºC – 59ºC	2 – 2.5	0.2 – 0.8	265 bp	No product
pufM_F2	pufM_WAW	50ºC – 59ºC	2 – 2.5	0.2 – 0.8	340 bp	No product
**<u>UniF</u>**	**<u>UniR</u>**	**<u>48ºC</u>** – 58ºC	2 – **<u>2.5</u>**	0.5 – **<u>0.8</u>**	145 bp	**<u>Amplification</u>**
UniF	pufM_WAW	48ºC – 58ºC	2 – 2.5	0.5 – 0.8	198 bp	No product

Table S3. Oceanographic context of the samples used in this study.

A. Open Ocean samples from the Malaspina Expedition (all collected at 3 m deep)

Sample	Date	Oceanic region	Latitude	Longitude
MP0311	4th January 2011	South Atlantic	-7.28	-29.32
MP0778	5th February 2011	South Atlantic	-33.23	15.34
MP1176	7th March 2011	Indian Ocean	-29.57	96.41
MP1421	25th March 2011	South Australian Bight	-40.55	142.49
MP1517	20th April 2011	South Pacific	-23.38	-178.21
MP1672	29th April 2011	South Pacific	-3.41	-169.46
MP1857	14th May 2011	North Pacific	21.89	-155.66
MP2243	2nd June 2011	North Pacific	10.76	-102.44

B. Surface coastal samples from the Blanes Bay Microbial Observatory (41°40′N, 2°48′E), in the NW Mediterranean Sea.

Sample	Date
BL110208	8th February 2011
BL110412	12th April 2011
BL110615	15th June 2011
BL110802	2nd August 2011
BL111129	29th November 2011
BL120214	14th February 2012
BL120411	11th April 2012
BL120620	20th June 2012
BL120807	7th August 2012

Table S4. Number of amplicon sequence variants (ASVs), or predicted number of genes in the metagenomic assay, retrieved by each method and environment.

	**ASVs**			
**Assays**	**Total (nº ASVs)**	**Open Ocean (Malaspina Exp)**	**Coastal (BBMO)**	**Common to both environments**
pufMF / pufM_WAW	418	252	180	14
pufMF_Y / pufM_WAW	1904	1397	563	56
UniF / UniR	1294	819	518	43
Metagenomic Assay	238	176	62	-

Table S5. Mantel test and Procrustes test statistics. Correlation in the Procrustes test refers to the correlation in a symmetric Procrustes rotation. The Mantel statistic is based on Pearson's product-moment correlation. Both tests were performed with 999 permutations.

			Mantel Test		Procrustes Test		
			**Mantel R**	**Significance**	**m_{12} squares**	**Correlation**	**Significance**
pufMF/pufM_WAW	*vs.*	pufMF_Y/pufM_WAW	0.8275	$p < 0.001$	0.0700	0.9643	$p = 0.001$
pufMF/pufM_WAW	*vs.*	UniF/UniR	0.7204	$p < 0.001$	0.2043	0.8920	$p = 0.001$
pufMF_Y/pufM_WAW	*vs.*	UniF/UniR	0.7938	$p < 0.001$	0.0728	0.9629	$p = 0.001$

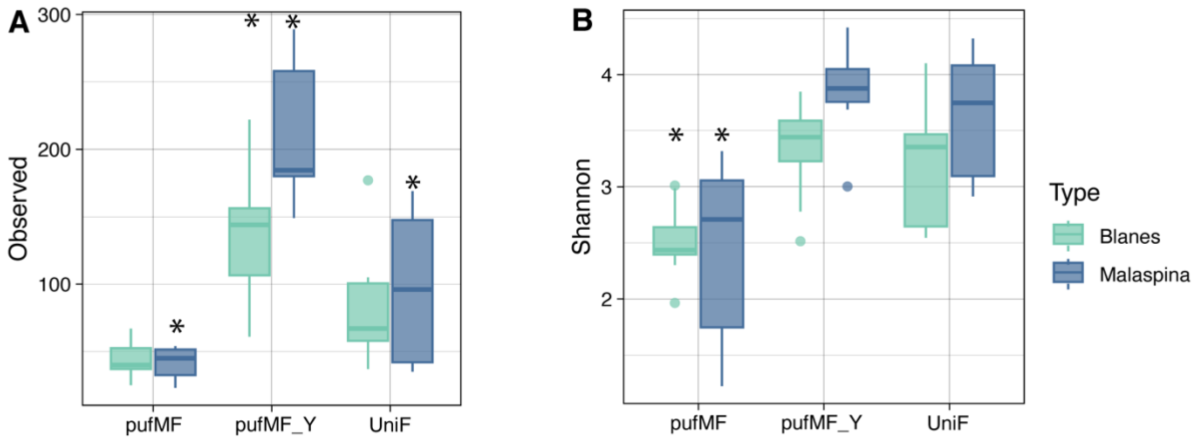

Figure S1. Alpha diversity as observed richness (A) and Shannon index (B) for each primer combination. * Asterisks indicate groups that are statistically different from the others, after an analysis of variance and a post-hoc Tukey test (* $p < 0.05$).

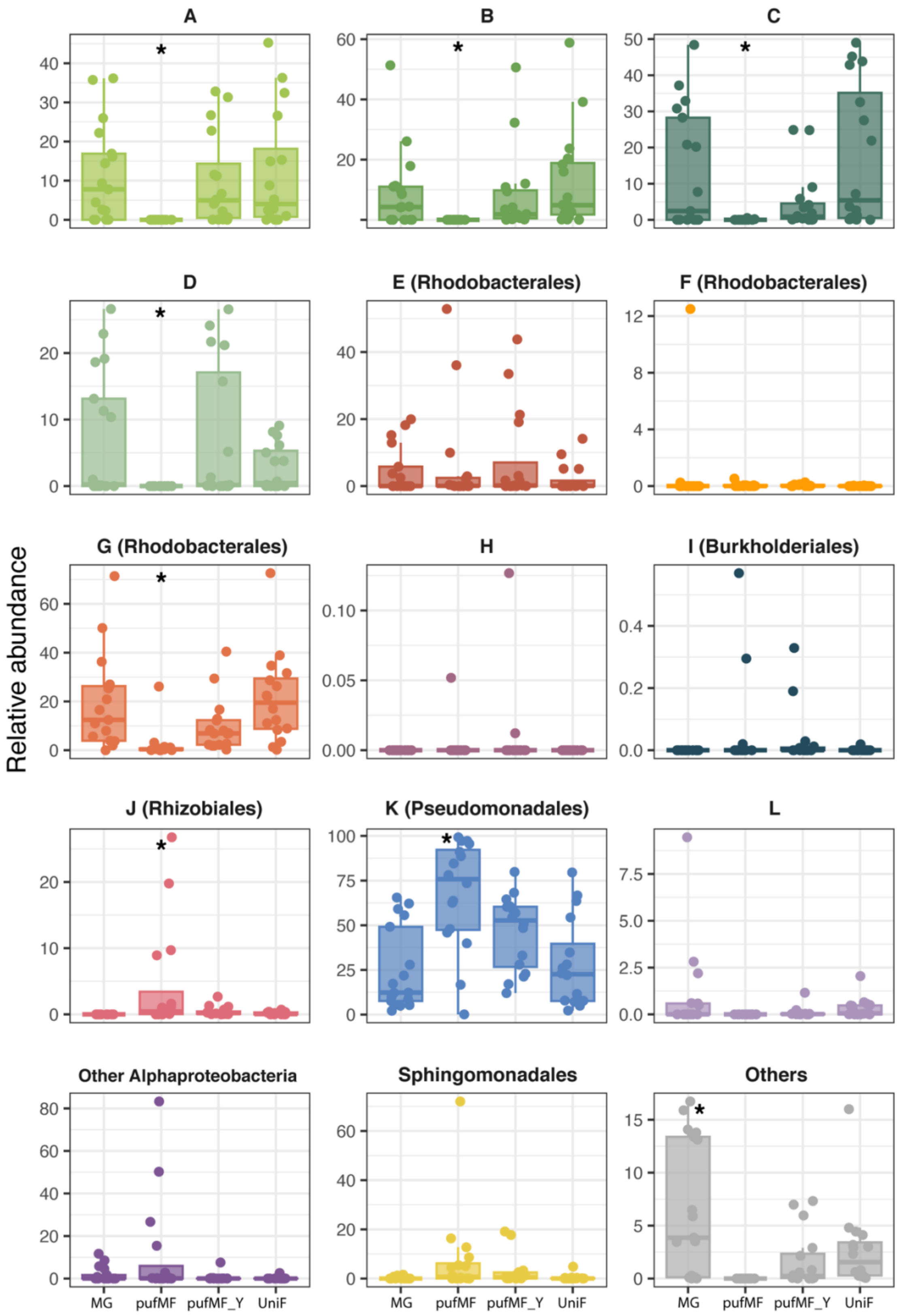

Figure S2. Relative abundance of the different taxonomic groups, retrieved in each amplicon approach and from the metagenomes. Samples of coastal and open ocean studies are pooled together. *Asterisks indicate groups that are statistically different from the others, after an analysis of variance and a post-hoc Tukey test (* p<0.05). MG: metagenomes. The amplicon approaches are identified with the forward primers.

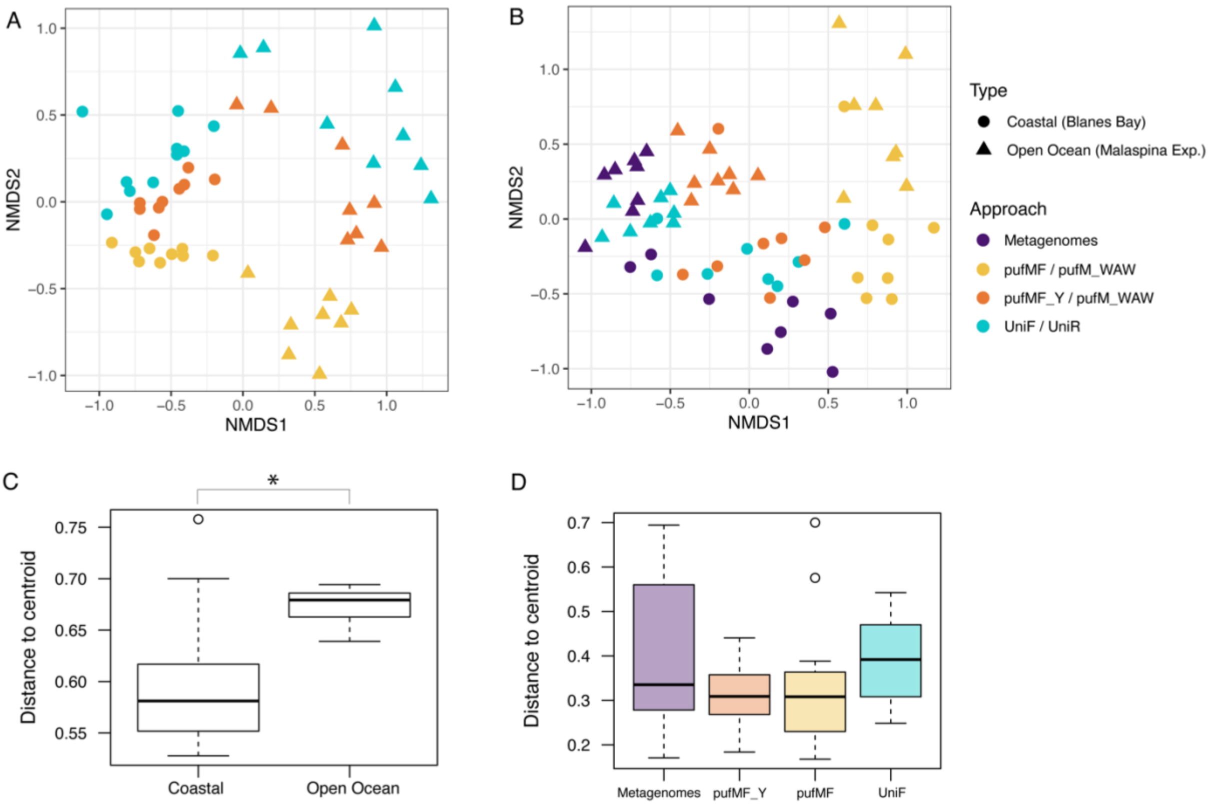

Figure S3. A) Non-metric multidimensional scaling (NMDS) of samples from the three amplicon approaches based on Bray-Curtis distances. B) NMDS of samples from the amplicon and the metagenomic approaches. This ordination is based on the abundance of the taxonomic groups instead of the abundance of ASVs (for the amplicon approaches) or contigs (for the metagenomes). Samples are color-coded based on the approach (see legend), and the type of environment (coastal and open ocean) is represented by circles and triangles respectively. C) Distance to centroids of coastal and open ocean environments samples (* p<0.0001), and D) Distance to centroids of the different amplicon and metagenomic approaches (p=0.059) after analysis with the *betadisper* and *permutest* functions.

Supplementary Information 1.

PCR conditions for primers pufMF_Y/pufM_WAW and UniF/UniR

Partial amplification of the *pufM* gene was performed with forward primer pufMF_Y and reverse primer pufM_WAW, each at 0.8 µM, and a final concentration of $MgCl_2$ of 2.5 mM. PCR conditions started with an initial denaturation step at 95 °C for 2 min, followed by 35 cycles at 95 °C (30s), 51°C (30s), 72 °C (40s) and a final elongation step at 72 °C for 10 min. PCR conditions for primers UniF and UniR were the following: an initial denaturation step at 95 °C for 2 min and 35 cycles at 95 °C (30s), 48°C (30s), 72 °C (40s) and a final elongation step at 72 °C for 10 min. The concentration of primers was 0.8 µM and the concentration of $MgCl_2$ was 2.5 mM.

Inferring amplicon sequence variants (ASVs) with the DADA2 pipeline:

The dataset from each combination of primers was processed separately in DADA2 v1.10 (Callahan et al., 2016). Sequences from primers pufMF and pufM_WAW were already analyzed in Auladell et al., (2019) and Gazulla et al., (2022), for the BBMO and the Malaspina Expedition samples respectively. In this study, we repeated the analyses with DADA2 for the 17 samples with the following parameters maxEE = c(2,2), trunclen = c(200,160). We kept 85% of total reads (mean 21311, min 8724, max 48334) after removing chimeras.

DADA2 parameters for the sequences obtained with primers pufMF_Y/pufM_WAW were maxEE = c(2,2), trunclen = c(190,120). After filtering we kept 83% of the total reads (mean 78047, min 26061, max 124270). Finally, for primers UniF/UniR DADA2 parameters were maxEE = c(2,2), trunclen = c(125,100) (the amplicon generated with this primer set is shorter). We kept 97% of total reads (mean 12319, min 1131, max 57444). Sample BL110412 had very low read counts (103) and was discarded in further analyses.

Supplementary Information 2.

Malaspina Expedition metagenomic analysis

A total of 76 DNA samples from 11 stations of the global tropical and sub-tropical oceans (water filtered through 3.0 and 0.22 µm polycarbonate filters) were sequenced on an Illumina HiSeq 2000 platform (2 x 101 bp) at the Centre Nacional d'Anàlisi Genòmica (CNAG, Barcelona, Spain). Raw reads were trimmed in trimmomatic 0.38 (Bolger et al., 2014) with options ILLUMINACLIP:2:30:10 LEADING:3 SLIDINGWINDOW:4:20 MINLEN:45. Clean reads were assembled in megahit 1.1.3 (Li *et al*., 2016) with options --presets meta-large and --min-contig-len 500. Assembled contigs were annotated in prokka 1.14.6 for gene prediction (Seeman, 2014), clusters of orthologous groups (COGs), Enzyme Commission numbers (EC) and gene product name. Additionally, predicted genes were annotated for protein families' domains (PFAM v34, El-Gebali *et al*., 2019) using HMMER 3.33 (Eddy, 2011), the Kyoto Encyclopedia of Genes and Genomes Orthologs (KEGG KO) release v98.0 using kofamscan 1.3.0 (Aramaki *et al*., 2019), and carbohydrate active enzymes (CAZyme) using HMMER 3.3 against dbCAN v10 (Yin *et al*., 2012).

Malaspina gene catalog

All predicted genes longer than 100 bp were pooled and clustered in cd-hit-est 4.6.1 (Li and Godzik, 2006) with options -c 0.95 -G 0 -aS 0.9 -g 1 -r 1 -d 0 -s 0.8. Clean reads were back-mapped to the catalog with bowtie2 2.2.9 (Langmead and Salzberg, 2012) and alignments were filtered with samtools 1.3.1 (Li *et al*., 2009) with options -q 10 -F 260. Reads mapping to catalog genes were counted in htseq-count 0.10.0 (Anders *et al*., 2015) with options --nonunique all to build gene profiles per sample. Counts were normalized by gene length in kbp and then normalized by the median abundance of 10 single copy COGs to obtain a proxy to per-cell number of gene copies as in Salazar *et al*., (2019).

Supplementary Information 3.

Statistical analyses

Data processing, statistical analyses and figures were performed in R v4.2.0 (R Core Team 2022). Alpha diversity values were calculated with *estimate_richness* function from *phyloseq* (McMuride and Holmes, 2013). The taxonomic composition of samples was represented with packages *phyloseq* (McMuride and Holmes, 2013) and *ggplot2* (Wickham, 2016), and we performed several statistical tests: we tested whether there were statistical differences between the taxonomic composition of samples retrieved with each primer combination using *adonis2* function (permutational multivariate analysis of variance), with Bray-Curtis distance matrices and 999 permutations. Also, for each taxonomic group, we performed analyses of variance and post-hoc Tukey tests (*aov* and *TukeyHDS* functions from *stats* package, version 3.6.2) to examine the relative abundance variation observed with each primer combination and with metagenomics.

For the community structure analyses we calculated Bray-Curtis distances with *vegdist* function, and Mantel correlations were performed with *mantel* function (999 permutations). We performed Procrustes tests to the three possible combinations between the three amplicon approaches (pufMF/pufM_WAW *vs.* pufMF_Y/pufM_WAW, pufMF/pufM_WAW *vs.* UniF/UniR and pufMF_Y/pufM_WAW *vs.* UniF/UniR). The data was Hellinger-transformed using *decostand* function and we calculated the m_{12} statistic using *procrustes* function. The significance of the m_{12} statistic was then tested with the *protest* function and 999 permutations. All the functions mentioned in this paragraph are from *vegan* package (Oksanen, 2022). Finally, the ordination plots were based on Bray-Curtis distances and calculated and represented with *ordinate* and *plot_ordination* from *phyloseq* (McMuride and Holmes, 2013). We used *betadisper* and *permutest* functions, both from *vegan* (Oksanen, 2022), to test the dispersion between samples from different environments (coastal *vs* open ocean) and assays (the three amplicon approaches and the metagenomic approach).

References:

Anders, S., Pyl, P.T., Huber, W., (2015). HTSeq—a Python framework to work with high-throughput sequencing data. *Bioinformatics* 31, 166–169. https://doi.org/10.1093/bioinformatics/btu638

Aramaki, T., Blanc-Mathieu, R., Endo, H., Ohkubo, K., Kanehisa, M., Goto, S., Ogata, H., (2019). KofamKOALA: KEGG ortholog assignment based on profile HMM and adaptive score threshold. *Bioinformatics* 1–2. https://doi.org/10.1093/bioinformatics/btz859

Béjà, O., Suzuki, M.T., Heidelberg, J.F., Nelson, W.C. Preston, C.M., Hamada, T., Elsen, J.A., Fraser, C.M., DeLong, E.F. (2002) Unsuspected diversity among marine aerobic anoxygenic phototrophs, *Nature*, 415, no. February, pp. 630–633, 2002.

Bolger, A.M., Lohse, M., Usadel, B., (2014). Trimmomatic: a flexible trimmer for Illumina sequence data. *Bioinformatics* 30, 2114–2120. https://doi.org/10.1093/bioinformatics/btu170

Eddy, S.R., (2011). Accelerated profile HMM searches. *PLoS Computational Biology* 7, e1002195, 1–16. https://doi.org/10.1371/journal.pcbi.1002195

El-Gebali, S., Mistry, J., Bateman, A., Eddy, S.R., Luciani, A., Potter, S.C., Qureshi, M., Richardson, L.J., Salazar, G.A., Smart, A., Sonnhammer, E.L.L., Hirsh, L., Paladin, L., Piovesan, D., Tosatto, S.C.E., Finn, R.D., (2019). The Pfam protein families database in 2019. *Nucleic Acids Research* 47, D427–D432. https://doi.org/10.1093/nar/gky995

Langmead, B., Salzberg, S.L., (2012). Fast gapped-read alignment with Bowtie 2. *Nature Methods* 9, 357–359. https://doi.org/10.1038/nmeth.1923

Li, D., Luo, R., Liu, C.M., Leung, C.M., Ting, H.F., Sadakane, K., Yamashita, H., Lam, T.W., (2016). MEGAHIT v1.0: A fast and scalable metagenome assembler driven by advanced methodologies and community practices. *Methods* 102, 3–11. https://doi.org/10.1016/j.ymeth.2016.02.020

Li, H., Handsaker, B., Wysoker, A., Fennell, T., Ruan, J., Homer, N., Marth, G., Abecasis, G., Durbin, R., (2009). The Sequence Alignment/Map format and SAMtools. *Bioinformatics* 25, 2078–2079. https://doi.org/10.1093/bioinformatics/btp352

Li, W., Godzik, A., (2006). Cd-hit: A fast program for clustering and comparing large sets of protein or nucleotide sequences. *Bioinformatics* 22, 1658–1659. https://doi.org/10.1093/bioinformatics/btl158

McMurdie PJ, Holmes S (2013) Phyloseq: An R Package for Reproducible Interactive Analysis and Graphics of Microbiome Census Data. PLoS One 8:4 DOI: 10.1371/journal.pone.0061217.

Oksanen J, Simpson G, Blanchet F et al (2022) vegan: Community Ecology Package_. R package version 2.6-2, https://cran.r-project.org/package=vegan

Salazar, G., Paoli, L., Alberti, A., Huerta-Cepas, J., Ruscheweyh, H.-J., Cuenca, M., Field, C.M., Coelho, L.P., Cruaud, C., Engelen, et al., (2019). Gene Expression Changes and

Community Turnover Differentially Shape the Global Ocean Metatranscriptome. *Cell* 179, 1068-1083.e21. https://doi.org/10.1016/j.cell.2019.10.014

Seemann, T., (2014). Prokka: Rapid prokaryotic genome annotation. *Bioinformatics*. https://doi.org/10.1093/bioinformatics/btu153

Wickham H (2016) ggplot2: Elegant Graphics for Data Analysis. Springer-Verlag New York. https://ggplot2.tidyverse.org

Yin, Y., Mao, X., Yang, J., Chen, X., Mao, F., Xu, Y., (2012). dbCAN: a web resource for automated carbohydrate-active enzyme annotation. *Nucleic Acids Res* 40, W445-51. https://doi.org/10.1093/nar/gks479

Yutin, N., Suzuki, M.T., Béjà, O., (2005). Novel Primers Reveal Wider Diversity among Marine Aerobic Anoxygenic Phototrophs. *Applied and Environmental Microbiology* 7, 12, p. 8958-8962. doi:10.1128/AEM.71.12.8958–8962.2005
